# Hearing and Vision Impairment in Older Adults: Implications for Cognitive, Mental, and Brain Health

**DOI:** 10.1093/geroni/igab046.593

**Published:** 2021-12-17

**Authors:** Jennifer Deal, Frank Lin, Bonnielin Swenor

## Abstract

Sensory impairment impacts over 55% of Americans aged 60 years or older and may have important downstream consequences for the cognitive health of older adults. This session will present evidence for a relationship between sensory impairment and accelerated cognitive decline, increased risk of incident dementia, and increased mental and physical fatigability from two observational cohort studies. Additionally, this session will investigate the possible nature of these relationships. It may be that sensory impairment is a marker of dementia-related pathological changes in the brain, with potential ramifications for risk prediction and stratification. Alternatively, sensory impairment may have a direct impact on the aging brain, a potential causal mechanism liking sensory impairment and brain health, with implications for disease prevention. As part of this session, we will present evidence for associations between central auditory processing, a potential risk marker, and brain volumes measured with structural magnetic resonance imaging (MRI), and retinal vasculature density, as measured with optical coherence tomography. We will conclude by describing associations between age-related macular degeneration, a leading cause of blindness, and neurocognitive test performance and regional changes in brain atrophy and connectivity.

